# Giant ulcerative lesion on the upper back: using a differential diagnosis to formulate a clinical approach

**DOI:** 10.1590/S1679-45082016AI3405

**Published:** 2016

**Authors:** Ryan David Wagner, Harrison Phu Nguyen, Stephen Keith Tyring

**Affiliations:** 1 Baylor College of Medicine, Houston, Texas, USA.; 2 University of Texas Medical School at Houston, Houston, Texas, USA.

A 57-year-old white man with no significant past medical history presented to the county hospital emergency room with complaints of increasing fatigue and lightheadedness over the past year. Upon further questioning, he revealed a large ulcerative lesion on his upper back that he reported started as a small ulcer and progressed to its current size over a 16-year period. The patient had not sought any medical attention throughout this time.

**Figure 1:**
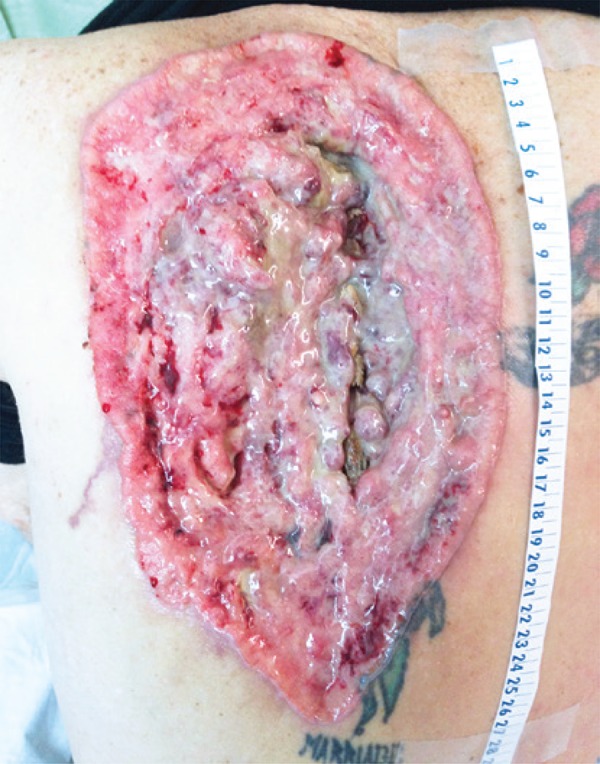
Giant ulcerative basal cell carcinoma of the upper back measuring 26cmx16cm with exposure of the paravertebral musculature and thoracic spinous processes. The patient had a tattoo on his back since adolescence, before the development of the lesion

The patient had no known history of malignancy, immunosuppressive conditions, autoimmune disorders, exposure to communicable diseases, or travel outside of the United States. His vital signs were within normal limits. Physical examination revealed a 26cmx16cm ulcerative lesion spanning the T1 through T8 vertebral bodies with exposure of the spinous processes and paravertebral musculature, which was most prominent at the level of T5. The lesion contained punctate areas of bleeding, granulation tissue, and copious serous drainage. The boarders were clearly defined and without satellite lesions (Figure 1). Other than pallor of the skin, the remainder of the physical examination, including a full neurological assessment, was unremarkable. In the emergency room, a computed tomography scan of the chest/abdomen/pelvis was performed and two individual punch biopsies of the ulcer bed were taken. The computed tomography scan showed erosion of the thoracic spinous processes but no evidence of metastatic disease. A complete blood count revealed a hemoglobin and white blood count of 4.6g/dL and 6.9 cells x 10^3^/µL, respectively. On admission the patient was transfused for his symptomatic anemia and started on ferrous sulfate.

**Figure 2:**
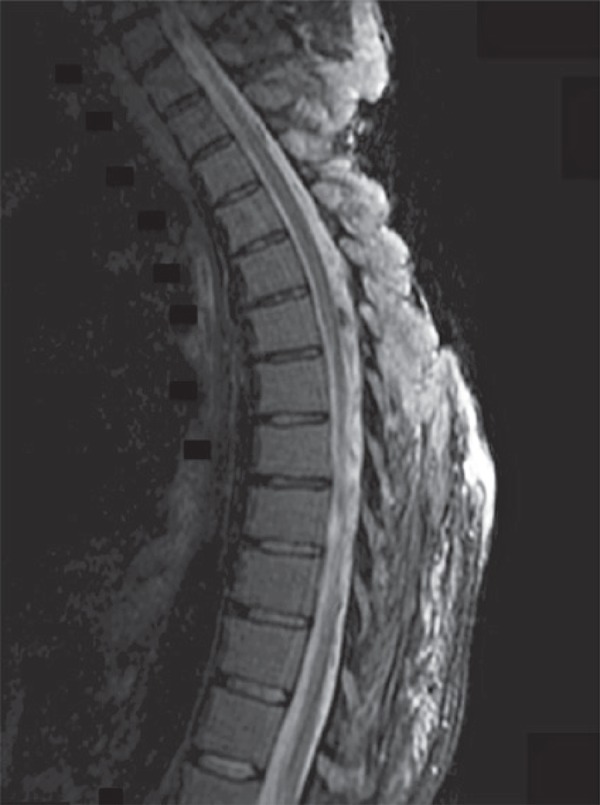
Midline sagittal view of the spine using magnetic resonance imaging demonstrating absence of spinal cord involvement

**Figure 3:**
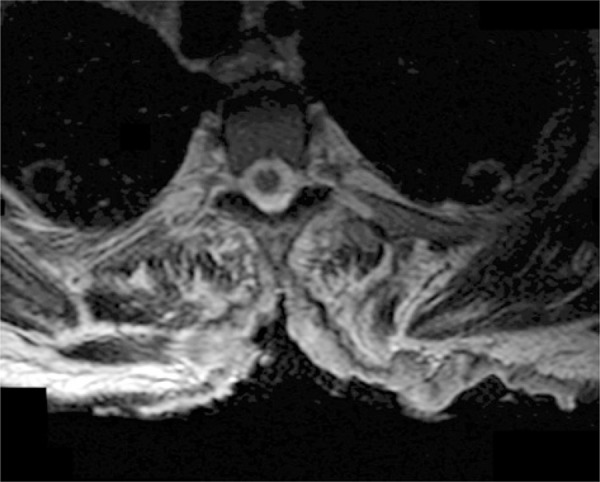
Axial view of the spine at T5 using magnetic resonance imaging demonstrating absence of spinal cord involvement

Despite the absence of neurological signs on physical exam, the magnetic resonance imaging of the back was required to assess for spinal cord involvement. Even without a pathological diagnosis, invasion of the spinal cord required urgent management. Dexamethasone was given until magnetic resonance imaging results confirmed the absence of spinal cord involvement (Figures 2 and 3).^1^ In addition, the until magnetic resonance imaging provided a more detailed picture of the depth of invasion and the local extension than the original computed tomography scan.

At this juncture, the proper formulation of a differential diagnosis is critical for guiding the next steps in management. The most likely pathogenic processes underlying cutaneous ulcers are immune-mediated, infectious, and neoplastic, although ulcers can also develop secondary to chronic venous or arterial insufficiency.^2^ Pyoderma gangrenosum, which is associated with a host of autoimmune diseases, including inflammatory bowel disease and rheumatoid arthritis, can often bear a similar ulcerative morphology, but without other co-morbidities and no symptoms such as abnormal bowel habits or joint pain. The diagnosis of pyoderma gangrenosum occurring independently is unlikely.^3^ For an infectious process, the differential diagnosis would include Buruli ulcer, which is focally endemic in Sub-Saharan Africa and is caused by *Mycobacterium ulcerans* ; phagedenic ulcer, a polybacterial infection with higher incidence in tropical regions; and necrotizing fasciitis caused by *Gram-* positive cocci.^4,5^ Of these infections, necrotizing fasciitis is associated with high fever and rapid progression. Taken together with the patient’s negative travel history, the absence of both fever and leukocytosis suggested a non-infectious disease process, and thus, empiric antibiotic treatment and bacterial cultures were not indicated. A vascular etiology was also unlikely given the location of the lesion and the absence of any prior trauma or radiation to that area.

After 2 days, histopathological results of the punch biopsies returned, with both specimens consistent with ulcerated basal cell carcinoma. The patient was given instructions for wound care, provided with supplies, and discharged with infectious disease, radiation oncology, and physical therapy referrals. An outpatient bone biopsy was ordered to assess for suspected osteomyelitis. The image-guided biopsy of the T3 spinous process confirmed acute osteomyelitis with a Gomori methenamine silver stain negative for fungal elements and an acid-fast bacilli stain negative for acid-fast organisms. After the multidisciplinary tumor board review excluded the possibility of surgical excision due to the wide extension of the lesion, radiation therapy was planned for management. Radiation therapy has previously shown efficiency for reducing the size of the target lesion and for symptom palliation in non-melanoma skin cancers using a 0-7-21 day regimen.^6^
