# Supplementary material for: Giant ulcerative lesion on the upper back: using a differential diagnosis to formulate a clinical approach
Source: Einstein (Sao Paulo). 2016 Jan-Mar;14(1):106–7. doi: 10.1590/S1679-45082016AI3405 (PMC4872926; doi:10.1590/S1679-45082016AI3405)
Supplement: Translation

# Translation: Lesão ulcerativa gigante no alto do dorso: diagnóstico diferencial para formulação de abordagem clínica

**Authors:** Ryan David Wagner, Harrison Phu Nguyen, Stephen Keith Tyring

**Affiliations:** 1 Baylor College of Medicine, Houston, Texas, USA.; 2 University of Texas Medical School at Houston, Houston, Texas, USA.

Homem branco, 57 anos, sem histórico médico significante, admitido no serviço de emergência queixando-se de fadiga aumentada e tontura iniciada há 1 ano. Durante a consulta, o paciente mencionou extensa lesão ulcerativa no alto do dorso, que iniciou como pequena úlcera e progrediu ao longo de 16 anos. Durante esse período, o paciente não procurou tratamento.

Não havia histórico de malignidade, condições imunossupressoras, exposição a doença contagiosa, ou relato de viagem para fora dos Estados Unidos. Os sinais vitais estavam dentro dos padrões normais. O exame físico revelou lesão ulcerativa medindo 26cmx16cm, abrangendo os corpos vertebrais de T1 a T8, com exposição dos processos espinhosos e musculatura paravertebral mais proeminente no nível de T5. A lesão continha áreas de sangramento pontilhados, tecido granulado e drenagem copiosa de seroma. As margens estavam bem definidas e sem lesões satélites (Figura 1). Além da palidez da pele no restante do exame físico, que incluiu avaliação neurológica completa, não foram observados outros fatores significantes. Na emergência, realizou-se tomografia computadorizada do tórax/abdômen, pélvis, além de duas biópsias individuais por punção do leito da úlcera. A tomografia computadorizada mostrou erosão dos processos espinhosos torácicos, porém não havia evidência de doença metastática. O hemograma relevou hemoglobina e leucócitos de 4,6g/dL e 6,9 células x 10^3^/µL, respectivamente. Na internação, o paciente recebeu transfusão devido à sua anemia assintomática e iniciou terapia com sulfato ferroso.

**Figura 1:** Carcinoma basocelular gigante ulcerado no alto do dorso medindo 26cmx16cm com exposição de musculatura paravertebral e processos espinhosos torácicos. O paciente possuía tatuagem no dorso desde sua adolescência, antes do desenvolvimento da lesão

Apesar da ausência de sinais neurológicos no exame físico, foi solicitada ressonância magnética do dorso, para avaliar o envolvimento da coluna vertebral. Mesmo sem diagnóstico patológico, a invasão da coluna vertebral sinalizou necessidade de conduta de emergência. Foi administrada dexametasona até que os resultados da ressonância magnética confirmassem ausência de envolvimento da coluna vertebral (Figuras 2 e 3).^1^ Além disso, a ressonância mostrou quadro mais detalhado da profundidade da invasão e da extensão do local, do que os resultados da tomografia computadorizada.

**Figura 2:** Visão da linha média sagital da espinha, por meio de ressonância magnética, demostrando ausência do envolvimento do cordão espinhal

**Figura 3:** Visão axial de espinha em T5, por meio de ressonância magnética, demostrando ausência de envolvimento do cordão espinhal

Formular um diagnóstico diferencial apropriado é crucial para guiar os próximos passos da conduta. Os processos patogênicos mais prováveis de úlceras cutâneas subjacentes são imunomediados, infecciosos e neoplásicos, apesar de a úlcera também poder se desenvolver secundariamente à insuficiência venosa ou a arterial crônicas.^2^ A piodermite gangrenosa, que é associada como hospedeira de doenças autoimune, incluindo doença inflamatória intestinal e artrite reumatoide, pode normalmente carregar morfologia ulcerativa similar, porém sem outras comorbidades e sintomas, como hábitos intestinais anormais ou dores articulares. O diagnóstico de piodermite gangrenosa de modo independente é improvável.^3^ Para um processo infecioso, o diagnóstico diferencial deve incluir úlcera de Buruli, que é focalmente endêmica na África Subsaariana e causada por *Mycobacterium ulcerans* ; úlcera fagedênica, infecção polibacteriana com alta incidência em regiões tropicais; e fasciite necrosante, causada por cocos *Gram* -positivos.^4,5^ Dessas infecções, a fasciite necrosante é associada com febre alta e progressão rápida. Devido ao não histórico de viagem do paciente, a ausência de febre e a leucocitose sugeriram processo de doença não infeciosa e, portanto, tratamento antibiótico empírico e culturas bacterianas não foram indicados. Também era improvável um etiologia vascular, dada a localização da lesão e a ausência de qualquer trauma anterior ou radiação na área.

Depois de 2 dias, os resultados histopatológicos das biópsias retornaram, e ambas as espécimes foram consistentes com carcinoma basocelular ulcerado. O paciente recebeu instruções para cuidar da ferida, teve suprimentos disponibilizados e recebeu alta, sendo encaminhado para tratamento de doença infeciosas, radiação oncológica e reabilitação física. Solicitou-se biópsia óssea, para avaliar suspeita de osteomelite. A biópsia guiada por imagem de processo espinhoso T3 confirmou osteomielite aguda com coloração de metenamina prata de Gomori negativa para elementos fúngicos, e coloração para a detecção de micobactérias negativa para bactérias ácido-álcool resistentes. Após exclusão da ressecção cirúrgica do tumor pela equipe multidisciplinar avaliadora, devido à sua extensão, foi agendada a radioterapia. A radioterapia já se mostrou efetiva para reduzir o tamanho de lesões e também para alívio dos sintomas em câncer de pele não melanoma, utilizando regime de 0-7-21 dias.^6^
